# Dynamic ultrasound assessment of pneumothorax extension: a comparison with computer tomography

**DOI:** 10.1186/1757-7241-20-S1-O10

**Published:** 2012-03-22

**Authors:** NP Oveland, HM Lossius, K Wemmelund, P Stokkeland, L Knudsen, E Sloth

**Affiliations:** 1 Department of Research and Development, Norwegian Air Ambulance Foundation, Droebak, Norway; 2 Department of Anesthesia and Intensive care medicine, Stavanger University Hospital, Stavanger, Norway; 3 Institute of Clinical Medicine, Aarhus University, Aarhus, Denmark; 4 Department of Radiology, Stavanger University Hospital, Stavanger, Norway; 5 Department of Anesthesia and Intensive care medicine, Aarhus University Hospital, Aarhus, Denmark

## Background

In trauma patients, ultrasound (US) is twice as sensitive as supine chest x-ray (CXR) in detecting occult pneumothorax (OPTX)[1]. The US “lung point” sign (LP) is 100% specific for PTX [2]. In spontaneous breathing patients, LP localization correlates with size and extension of OPTX [3], but uncertainty exists in patients on positive pressure ventilation (PPV).

## Objective

To compare LP identification using thoracic US and Computer Tomography (CT).

## Methods

Air was introduced into 5 hemithoraces (HTs) of 3 PPV porcine models. An anaesthesiologist experienced in US, identified LPs during the inspiratory phase and delineated the topography and extension of the PTX with subcutaneous needles. This was compared with the points where the lung detached from the inside of the chest wall identified by CT. The distance from sternum to the LP (S-LP) and PTX area were measured in two preset levels.

## Results

The total mean difference between US and CT in designation of 131 LPs were 6.8±7.1 mm (range 0-29.3 mm). The lateral limits of each PTX were collocated at different chest positions (ie, anterior, lateral and posterior) at 6.8±8.6, 6.4±6.1 and 7.3±6.4 mm, respectively. A linear correlation was found between the S-LP distance and increasing PTX size in 9 out of 10 sets of measurements (Pearson coefficient ranged from 0.839 to 0.966, p≤0.05). An equal correlation was found with PTX area (Pearson coefficient ranged from 0.890 to 0.979 p≤0.05.

## Conclusion

US proved accurate in identifying the LP. PTX size correlated with the lateral LP position. US examination can guide clinical decisions on the patient’s need of a chest tube [3]. If PPV trauma patients with OPTX safely can be observed without tube thoracostomy is debated [4], but when chosen, we recommend close observation with repeated use of US.
